# Survey of Decomposition-Reconstruction-Based Hybrid Approaches for Short-Term Traffic State Forecasting

**DOI:** 10.3390/s22145263

**Published:** 2022-07-14

**Authors:** Yu Chen, Wei Wang, Xuedong Hua, De Zhao

**Affiliations:** 1 School of Transportation, Southeast University, Nanjing 210096, China; 230228902@seu.edu.cn (Y.C.); huaxuedong@seu.edu.cn (X.H.); zhaode@seu.edu.cn (D.Z.); 2 Jiangsu Province Collaborative Innovation Center of Modern Urban Traffic Technologies, Nanjing 210096, China

**Keywords:** decomposition-reconstruction, traffic state forecasting, intelligent transportation system, predictability, interpretability

## Abstract

Traffic state prediction provides key information for intelligent transportation systems (ITSs) for proactive traffic management, the importance of which has become the reason for the tremendous number of research papers in this field. Over the last few decades, the decomposition-reconstruction (DR) hybrid models have been favored by numerous researchers to provide a more robust framework for short-term traffic state prediction for ITSs. This study surveyed DR-based works for short-term traffic state forecasting that were reported in the past circa twenty years, particularly focusing on how decomposition and reconstruction strategies could be utilized to enhance the predictability and interpretability of basic predictive models of traffic parameters. The reported DR-based models were classified and their applications in this area were scrutinized. Discussion and potential future directions are also provided to support more sophisticated applications. This work offers modelers suggestions and helps to choose appropriate decomposition and reconstruction strategies in their research and applications.

## 1. Introduction

Short-term traffic state forecasting has always been one of the hotspots in the field of transportation research and a prerequisite for the successful operation of intelligent transportation systems (ITSs). Interest in it may stem from the growing demand for developing user-friendly platforms, such as advanced traffic management systems (ATMSs) and advanced traveler information systems (ATISs). Accurate and real-time traffic state forecasting helps to proactively manage the transportation network and enhance the travel experience for commuters [1,2,3].

In recent years, an increasing number of detection sensors have been deployed in transportation networks to detect traffic information, such as traffic flow and speed, in real time. Furthermore, the automatic fare collection systems for public transit collect a large amount of data, such as passenger flows. These data can be used for traffic counting and analysis, such as road traffic noise mapping computations or traffic pattern recognition [4,5].

The objective of short-term traffic state prediction is to estimate how traffic parameters, such as flow and speed, will evolve in the next few minutes or hours based on historical or observed traffic data. A large body of research has been published in this area over the last few decades. The approaches proposed by researchers for short-term traffic state prediction can be roughly divided into two categories: statistical approaches [6] and data-driven approaches [7,8,9].

Statistical approaches have been widely used in previous research for a well-developed theoretical framework and better interpretability, such as exponential smoothing (ES), Kalman filter (KF), and autoregressive integrated moving average (ARIMA).

Data-driven methods have attracted increasing popularity for two reasons: First, conventional statistical methods fail to capture the nonlinear features of traffic state series [10]. Second, the massively and widely deployed traffic sensors increase data volume and availability [11]. More applied data-driven methods include support vector regression (SVR), neural networks (NN) with various deep architectures, such as recurrent NNs (RNNs) and convolutional NNs (CNNs), and tree-based models, such as extreme gradient boosting (XGBoost). Data-driven models usually outperform statistical approaches in terms of accuracy; however, such models are generally black boxes with poor interpretability and are computationally inefficient [7].

Significant achievements have been made in the application of short-term traffic state prediction to alleviate traffic congestion. However, according to a review report by Vlahogianni et al. [2], several challenges still exist and are relatively understudied. Three of these challenging issues are: (a) how to discard the noise or improve the quality of probed traffic data used for forecasting, because a larger fraction of noise in the time series of the traffic parameters results in a more tedious development of forecasting models, and usually worse forecasting accuracy [2]; (b) how to cogently define and retrieve temporal characteristics and spatial dependencies of the traffic state, because the incorporation of these patterns may tangibly support the improvement of predictions [12]; and (c) how to improve the transparency of data-driven approaches or enhance the explanatory power of these models, as this information makes the forecasting model more robust and adaptable to the dynamically changing traffic environment [7].

Over the past 20 years or so, more than one hundred publications have used the decomposition-reconstruction (DR) strategy, also known as “divide-and-conquer” in some pieces of literature [13,14], to model the short-term traffic state. The concept of the DR strategy is mainly based on two assumptions: (a) predictability [15] (DR-based methods can be used to eliminate or suppress noise in traffic state sequences, remove outliers, and fill in missing values, in this way improving sequence smoothness and predictability) and (b) interpretability [16] (DR-based methods help identify intrinsic travel patterns (such as daily variations) in traffic state sequences that can be separated to enhance the explanatory power of the forecasting models).

Generally, DR processes provide a rich framework for modeling and analyzing time-varying traffic states (flow, speed, etc.). Various intrinsic travel patterns can be identified and extracted based on the different decomposition strategies used by researchers. For example, a wavelet transform (WT)-based strategy was used in [17] to extract various frequency features from the traffic flow sequences. Compared to benchmark non-decomposition methods (i.e., basic predictive models such as ARIMA and NNs) that are directly based on raw data without considering underlying patterns, DR procedures treat traffic count data such as traffic flow as a superposition of various components, such as periodic trend components and random fluctuating components. DR procedures aid in separating these components, thereby enhancing the stability and predictability of the data used for prediction [17]. In the literature reviewed, DR methods are usually reported to gain an improved prediction accuracy ranging from 5% to 80% compared to the corresponding benchmark non-decomposition methods (e.g., ANN and SVM [14]).

This paper presents a survey of different DR process strategies, including the Fourier transform (FT), WT, empirical mode decomposition (EMD), variational mode decomposition (VMD), and singular spectrum analysis (SSA), applied to short-term traffic state prediction over the past two decades. Other methods, such as structural time series (STS) and seasonal trend decomposition using LOESS (STL), were also surveyed. Although several review papers have been published on short-term traffic state prediction [1,2,6,7,8,9,12,18], to the best of the authors’ knowledge, no single review has been devoted to DR-based hybrid models in this field. This study aims to fill this gap and mainly focuses on how DR strategies have been utilized in the past 20 years to enhance the predictability and interpretability of basic predictive models of traffic parameters including flow, speed, travel time, passenger/freight volume, travel demand, and even accidents. Specifically, the range of transportation modes investigated in this study included highways/urban roads, public transit (bus and metro), railways, and aviation. The scope of the investigation overall followed that of a previous study [7], in which papers to predict traffic parameters including flow, passenger volume, speed, density, and occupancy were surveyed concurrently. However, this study extended the scope of the survey presented in Ref. [7]. Models for the prediction of bus travel time, passenger flow in metro systems, and air and rail passenger demands were also considered. These prediction applications, including short-term forecasting of traffic flow, speed, and travel time of highways or city roads, passenger demand for air or railways, and passenger volume in public transit systems, although slightly different in the use of data types, belong to the same domain of traffic-counting analysis and therefore were surveyed concurrently in this study. The survey of this paper sheds light on the current state of practice across multiple studies and inspires us to discuss issues that require further research.

The remainder of this paper proceeds as follows. Section 2 contains a general theoretical overview of DR procedures. Section 3 reviews DR-based short-term traffic state forecasting models categorized according to the types of decomposition strategies. We then discuss in Section 4 several limitations in the application of these DR procedures and attempt to provide a set of insights for further research. Finally, Section 5 summarizes the study.

## 2. Decomposition-Reconstruction-Based Short-Term Traffic State Forecast: An Overview

**Figure 1:**
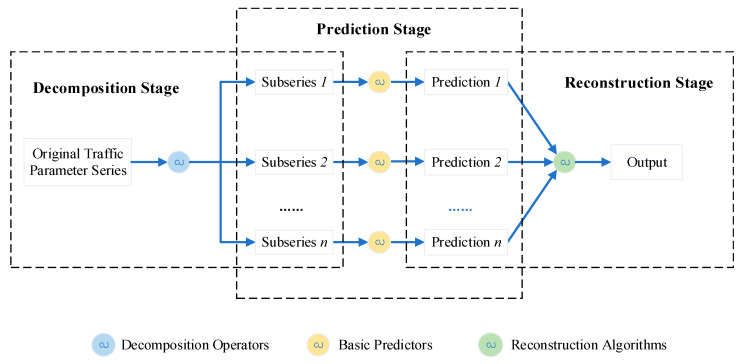
General procedure of decomposition-reconstruction or divide-and-conquer.

Generally, DR-based hybrid models for short-term traffic state prediction share a similar framework, despite using different decomposition or reconstruction strategies, as shown in Figure 1. This framework comprises three stages: decomposition, prediction, and reconstruction. In the decomposition stage, an original traffic parameter sequence is decomposed into several sub-sequences with different characteristics through the decomposition operator; in the forecasting stage, the aforementioned statistical methods and data-driven methods will provide their respective advantages to obtain the forecasting output, and in the reconstruction stage, the forecasting outputs of all sub-sequences are reconstructed into the final results.

Despite the clarity of the general procedure, several points should be noted:

(a) Input data can be either a single time series or panel data [19]. Panel data generally consist of multiple sequences of traffic parameters collected from multiple detection devices or locations, such as loop detectors, bus stops, or shared bicycle stations.
(b) The traffic parameter data used for decomposition are not strictly required to be complete and there may be missing or outlier values. DR procedures can be used to identify missing data or outliers in the original data and perform data correction [20,21].
(c) Various decomposition strategies have their strengths and weaknesses. In practical applications, there is no consistent conclusion regarding the decomposition strategy that can achieve the best prediction performance. From the published literature, most of the decomposition strategies adopted belong to one of the following types: FT, WT, EMD, VMD, SSA, STS, and STL. In addition, a few researchers have also tried the joint decomposition strategy, most commonly secondary decomposition (SD) [22,23], which means that after using one decomposition strategy to decompose the original data, another decomposition strategy is used for further decomposition of the decomposed sub-series to obtain more detailed traffic pattern information. However, it should be noted that the SD strategy significantly increases the computational complexity, and researchers need to carefully consider the balance between prediction accuracy and computational cost.
(d) Not all decomposed sub-series are necessarily fed into the predictor, and researchers select or reconstruct the components they consider valuable according to their expertise. For example, in some studies [24,25], the residual component obtained by decomposition was discarded to achieve denoising. As another example [26,27], researchers have used feature selection algorithms to obtain the most suitable components as inputs for basic predictors.
(e) In the prediction stage, the statistical or data-driven methods are selected by the modelers according to their expertise; however, it is not necessary to assign the same type of predictors for all components. A more general approach is to choose the most suitable algorithm according to the characteristics of the components (such as predictability or stationarity) [28,29]. In addition, in a few other studies [19], only one predictive model was established, in which all selected features were input simultaneously.
(f) In the reconstruction stage, researchers can either perform linear reconstruction, such as an equal-weighted summation of the results of each predictor [28], or nonlinear reconstruction, for example, adopting an SVM to achieve the optimally weighted combination of each component [30].

## 3. Decomposition-Reconstruction-Based Hybrid Models

In this section, we review DR-based short-term traffic state prediction models that use different decomposition strategies. Specifically, FT-based, WT-based, EMD-based, VMD-based, and SSA-based papers are reviewed in Section 3.1, Section 3.2, Section 3.3, Section 3.4, Section 3.5, respectively. Section 3.6 introduces the remaining two decomposition strategies: STS and STL.

**Figure 2:**
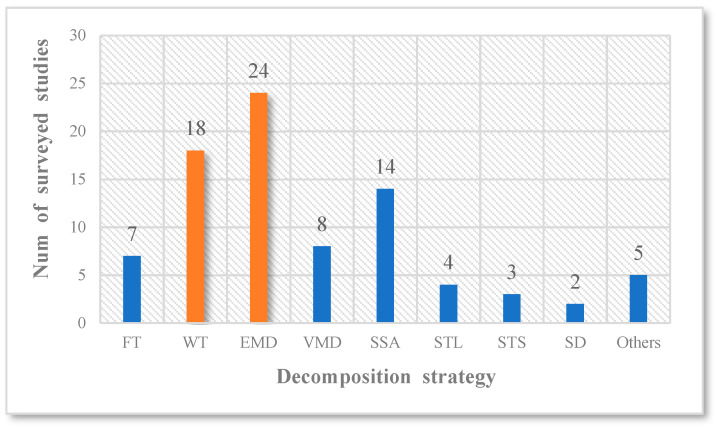
Statistics of decomposition strategies.

The statistics of the surveyed studies using various decomposition strategies (including their variants) are shown in Figure 2. It can be observed that the most used decomposition strategies are the EMD and WT methods, accounting for approximately half.

### 3.1. Fourier Transform

As one of the most popular transform methods, the FT has been commonly utilized in previous transportation research areas, including data abnormality detection [20], public parking space prediction [31], and dominant frequency extraction [32].

Typically, a traffic state time series can be viewed as a superposition of potential frequency components (e.g., daily and weekly), and a common intuition is that one can achieve better forecasting accuracy by incorporating such frequency-level information into their prediction models.

Peng et al. [33] developed a frequency-aware spatiotemporal network (FASTNet) for traffic flow forecasting. The spectrum of a traffic flow series that reflects certain travel patterns of passengers was obtained by applying the discrete Fourier transform (DFT). The traffic flow is then accurately predicted through a filtering mechanism based on frequency and spatiotemporal networks.

Chen et al. [34] adopted FT to decompose traffic flow into different components in terms of three aspects: periodicity and volatility, traveling purposes, and vehicle types. Therefore, time-series analysis and supervised learning were used to predict the different components according to their characteristics.

Luo et al. [35] used DFT to extract common trends in road traffic flow series and predicted them by extreme extrapolation. Chang et al. [36] proposed a novel data fusion method for travel time prediction and adopted FT for continuous parameterized modeling of the spatiotemporal pattern of spot speed (time-mean speed). Peeta et al. [20] presented an FT-based fault-tolerant mechanism to detect and correct data abnormalities owing to malfunctioning sensors for the seamless operation of an online architecture for active traffic control. Wang et al. [25] studied the noise removal problem of traffic flow sequences. They used a fast Fourier transform (FFT) method to denoise the noisy traffic flow signal and proposed a cross-validation-based adaptive cutoff frequency selection method (A-CFS) to determine the proper cutoff frequency, thereby effectively separating high-frequency noise from valuable information. Experiments were conducted using real-world traffic flow data obtained from a highway near Birmingham, UK, and the results demonstrated the competitiveness of the proposed method compared with two other commonly used denoising methods, WT and EMD.

### 3.2. Wavelet Transform and Its Variants

#### 3.2.1. Wavelet Transform

WT was developed based on the FT and provides a more powerful technique for multiresolution analysis of time series in both the time and frequency domains. In particular, WT is more effective than classical FT in analyzing nonstationary time-series data, and it can decompose the time series into several sub-sequences with different frequencies for independent analysis [37]. Discrete wavelet transform (DWT) and continuous wavelet transform (CWT) are its two basic forms; however, because traffic time series are discrete sequences, only DWT is suitable for short-term traffic forecasting [38].

**Figure 3:**
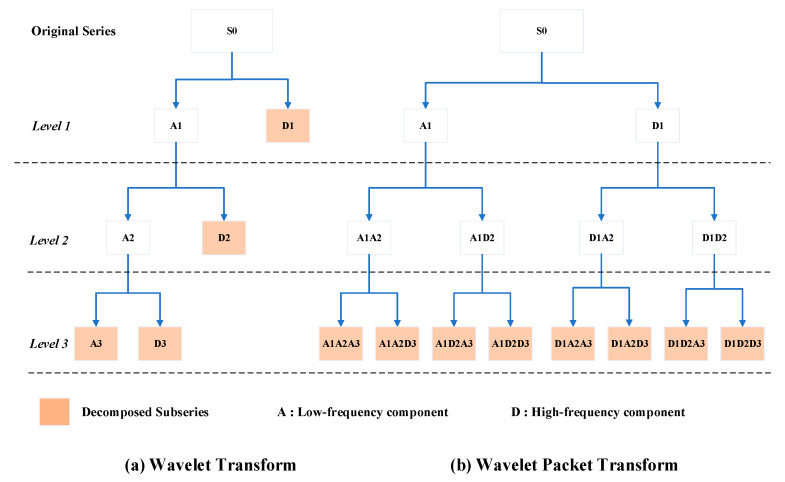
Schematic of wavelet decomposition and wavelet packet decomposition.

The fundamentals of WT are shown in Figure 3a. The previous-level sequence is decomposed into a detail sequence (high-frequency component) and an approximate sequence (low-frequency component) using a low- and high-pass filter for an independent analysis.

Xiao et al. [39] were some of the first scholars to incorporate WT into transportation research. They developed a short-term traffic speed forecasting framework that could eliminate noise due to random travel conditions (e.g., weather conditions), in which the wavelet denoising method was emphasized and analyzed. Jiang et al. [40] designed a novel nonparametric time-delay recurrent wavelet neural network for traffic flow prediction that adaptively integrated wavelets with neural networks. Xie et al. combined WT with a neural network [38] and the Kalman filter [24] for short-term traffic volume forecasting and discussed the differences between the two types of basis wavelets on the performance of WT-based models. Similar reports can also be found in [41] by Ghosh et al. In [41], the WT and Bayesian hierarchical methodology (BHM) collaborated to model short-term flow evolution at two intersections in the city center of Dublin.

Diao et al. [29] applied DWT to decompose the traffic volume sequence into an appropriate (low-frequency) component and several detailed (high-frequency) components, and employed a tracking model and a novel Gaussian process model to forecast the appropriate component and detail components, respectively. Moreover, the WT algorithm has been embedded to improve the performance of ARIMA [42], artificial NN (ANN) [19], least squares support vector machines (LS-SVMs) [43], CNN-RNN [44], and the multi-dimensional Taylor network model (MTN) [17] in short-term traffic flow forecasting. 

The implementation of WT is not limited to short-term traffic flow prediction and has also been reported in the forecasting of freeway travel time [45], urban traffic speed [46], urban rail transit ridership [47], passenger volume in subway systems [48], and real-time video traffic [49].

Practical applications from the previously reported works in the literature demonstrate that the WT method helps (a) acquire denoised historical traffic state measurements to improve predictability, and (b) identify intrinsic patterns in the traffic state and thus improve the interpretability of constructed forecasting models.

#### 3.2.2. Variants of Wavelet Transform

A variant of the WT is called wavelet packet transform (WPT). The principle of WPT is similar to that of the WT, except that WPT decomposes both the low- and high-frequency components. In general, the frequency band obtained through the DWT is more detailed than that of the WT. Figure 3b shows the fundamentals of WPT.

Jiang et al. [50] proposed a hybrid WPD–ACF method for short-term traffic flow forecasting, where the statistical autocorrelation function (ACF) was utilized for decomposition level determination in wavelet multiresolution analysis of traffic flow time series. Zhao et al. [22] adopted a secondary decomposition structure to predict passenger demand for China’s high-speed rails (HSRs). Specifically, the SSA method was first used to decompose the original data into one principal component and several detail components, and the principal component was further decomposed by WPD. The decomposed sub-series of the principal component was fed into the CNN for prediction, whereas the detailed components were modeled using SVM. The influence of WPD on the performance of the NN was also compared with other commonly used decomposition methods in [51].

According to published reports, WPD is usually more effective than WT for short-term traffic state forecasting. However, it should be noted that WPD has a higher computational complexity; thus, the general applicability of WPD requires further verification.

Furthermore, a few reports [37,43,52] have also pointed out that some defects exist in DWT that may make it unsuitable for further time-series analysis. Therefore, the stationary edition of the DWT, known as the stationary wavelet transform (SWT), was proposed by researchers to overcome these defects [37,43,53]. For instance, Dunne et al. [37] used precipitation as an exogenous variable and executed the SWT algorithm and neuro-wavelet methods to predict hourly traffic flow. Evaluation results at two dissimilar traffic sites in Dublin showed the generalizability and portability of the SWT. Boto-Giralda et al. [53] incorporated the SWT denoising process and the classification and pattern recognition capabilities of self-organizing fuzzy NNs (SFNNs) to analyze the decomposed traffic flow components independently. Experiments based on real-world data collected by four detectors located on interstate roads I-5, I-90, and I-405 (USA) demonstrated the outstanding performance of the proposed method.

### 3.3. Empirical Mode Decomposition and Its Variants

#### 3.3.1. Empirical Mode Decomposition

The classical empirical mode decomposition (EMD) method was first introduced by Huang et al. [54] at NASA as a key part of the Hilbert–Huang transform for analyzing nonlinear and nonstationary data. The EMD method can adaptively decompose a nonstationary signal into a finite and often small number of stationary intrinsic mode functions (IMFs) and a residue. Importantly, decomposed IMFs with different frequency bands (including the residue) can represent the physical meaning of reality [55]. The effectiveness of EMD has been demonstrated in extensive practice, including traffic engineering, for analyzing nonlinear and nonstationary processes.

Hamad et al. [56], for the first time, incorporated EMD into multilayer feedforward NNs with backpropagation for travel speed prediction. Experimental results based on real-world loop detector data obtained from I-66 in Fairfax, Virginia, showed that by decomposing the speed time series into its basic components via EMD, more accurate forecasts could be obtained. Wang et al. [57,58] and Kianifar et al. [59] performed similar studies by combining EMD with ARIMA and SVR, respectively, for highway speed prediction. Wang et al. [57] also explored the accuracy of the hybrid model under different traffic scenarios, including a work zone on Interstate I91 in Springfield, MA, and an on/off-ramp on Georgia State Route 400. Kianifar et al. [59] proposed a stacking ensemble learning method and tested its effectiveness on the Strategic Road Network (SRN1) dataset managed by the Highways Agency of England. Chen et al. [60] explored a novel bus travel time forecasting technique that combined EMD for speed data analysis and GM for travel time prediction. Their experiments at the Taiwan National Central University showed that the combined technique performed well.

Wei et al. [26] presented a hybrid EMD–NN approach for short-term passenger flow prediction in metro systems. This approach selects only the meaningful IMFs by applying Pearson product-moment correlation (PCC) and Kendall rank correlation (KRC) as inputs for back-propagation NNs (BPNs) while considering temporal factors as external inputs (e.g., the day of the week, the period of the day). A similar study was conducted by Moscoso-Lopez et al. [61], who created an optimal forecasting model for daily freight volume at ports. In their model, EMD transforms the time series into several simpler-to-predict sub-series, and permutation entropy (PE) identifies the complexity of the decomposed sub-series to aggregate the least complex ones and reduce the computational cost, whereas ANNs are applied to forecast the identified sub-series.

Although the effectiveness of EMD in short-term traffic state prediction has been proved by more works of literature [62,63,64], a few limitations of EMD have still been noticed, such as the “end effect” and “modal aliasing”, which may lead to misrepresenting of the decomposed IMFs [65,66]. Accordingly, Yang et al. [67] built a hybrid model including EMD and stacked auto-encoders (SAEs) and utilized a slope-based approach to address the “end effect” problem that existed in EMD. The proposed model was tested for traffic flow prediction using data from three freeways in the UK.

#### 3.3.2. Variants of Empirical Mode Decomposition

In view of the modal aliasing problem, several extended versions of EMD have been proposed, including ensemble empirical mode decomposition (EEMD) [68,69], complete ensemble empirical mode decomposition (CEEMD), and complete ensemble empirical mode decomposition with adaptive noise (CEEMDAN) [70,71]. These extended versions alter the distribution of the extreme points (places where a function takes on an extreme value) by adding noise to the original signal. The added noise signals cancel each other out through multiple integrated averaging processes, and the real signal components are gradually revealed in this process.

An EEMD-based hybrid model with a random vector functional link network (RVFL) as the basic predictor was proposed in [68] for short-term travel time forecasting. EEMD is first implemented to decompose the complex travel time series into several simpler IMFs, which are then modeled using the same number of RVFLs. The results were obtained using a linear combination of all RVFL outputs. In contrast to simple linear summation, Jiang et al. [30] employed SVM in short-term HSR passenger flow prediction experiments to achieve the final nonlinear reconstruction, in which particle swarm optimization (PSO) was used for model parameter calibration.

Zhang et al. [72] proposed an innovative hybrid approach, DeepEnsemble, for network-wide short-term traffic speed forecasting. The core steps of this approach include decomposing noisy traffic speed data using EEMD and constructing 3D tensors for CNN prediction. A case study on a large-scale urban expressway network in Beijing, China demonstrated the superiority of the proposed approach. Liu et al. [27] investigated the application of EEMD and deep belief networks (DBNs) for short-term traffic flow forecasting. After decomposing the traffic flow data into several IMFs and a residue with EEMD, a subset of essential features was extracted using the minimum redundancy maximum relevance (MRMR) method considering day properties and weather conditions. The DBNs were then used to predict each selected component. Validation experiments were conducted using data from the Portland–Vancouver metropolitan region.

Similar reports can be found in [28,64,66,73,74,75]. In [28], the approximate entropy (AE) method was used to evaluate the complexity of the decomposed components, and the echo states NN (ESNN), SVR, and ARMA were selected to predict the traffic flow components with high, medium, and low complexities, respectively. Chen et al. [73] slightly modified the EEMD method and adopted a quantum NN (QNN) for traffic flow forecasting. Chen et al. [74] and Tang et al. [75] used an ANN and fuzzy C-means NN (FCMNN) to predict the decomposed traffic flow series, respectively. Bao et al. [66] also involved a slope-based method to restrain the “end effect” phenomenon occurring during the shifting process of EEMD for air passenger traffic forecasting. To predict both the port cargo throughput and vessel traffic flow, Li et al. [64] further regrouped the high-frequency components decomposed by EMD and EEMD to achieve higher predictability.

Additionally, reports of short-term traffic forecasting based on CEEMDAN can also be found in [70,71,76]. Tian et al. [70] slightly improved the CEEMDAN algorithm and utilized permutation entropy (PE) to analyze the random properties of the decomposed IMFs. The kernel online sequential extreme learning machine (KOSELM) and ARIMA were then selected according to the different random properties of the IMFs for short-term traffic flow prediction at signalized intersections. Wang et al. [71] adopted the improved weighted permutation entropy (IWPE) to obtain new reconstructed components after decomposing the raw nonlinear and nonstationary highway traffic flow data using CEEMDAN. In the prediction stage, a grey wolf optimizer (GWO) is used to calibrate the least-squares support vector machine (LSSVM) established for each reconstruction component. Luo et al. [76] combined CEEMDAN and extreme gradient boosting (XGBoost) for lane-level traffic flow predictions.

### 3.4. Variational Mode Decomposition

The VMD method was initially developed by Dragomiretskiy et al. [77] in 2014 to provide a better solution to the decomposition problem. The VMD strategy addresses several limitations of previous techniques, such as the stationarity assumption in FT [78], basis wavelet and decomposition level selections in WT [50], and sensitivity to noise and sampling in EMD [54]. A notable strength of VMD was displayed in traffic engineering studies [79], and a body of experiments showed that VMD can provide valuable insights into the understanding of the intrinsic properties of traffic data [14].

Bing et al. [79] combined the VMD algorithm with a long short-term memory (LSTM) model for multistep ahead short-term traffic flow forecasting. Case studies proved that VMD outperformed EMD and WT in extracting trend information from traffic-flow data. Kim et al. [14] predicted the travel speed in urban networks in South Korea and analyzed the spectral and statistical properties of the decomposed traffic speed modes via VMD. Their experiment demonstrated that forecasting travel speed in urban networks becomes easier if the typical daily and commuting patterns can be explained through decomposed modes. Jin et al. [80] proposed a hybrid approach to short-term air passenger demand forecasting. VMD was first adopted to mitigate the complexity of the original data, and then the ADF test was executed to classify all the decomposed modes via VMD into stable and unstable sets. In the prediction stage, the ARMA and KELM models were employed to forecast the stable and unstable components, respectively. The final outputs were nonlinearly integrated using another KELM.

More experiments on short-term traffic flow prediction were conducted in [81,82], where VMD was combined with an ESN for prediction. VMD has also been applied to public transport forecasting [83,84]. Zhang et al. [83] applied VMD to enhance the forecasting of metro passenger volume. VMD decomposes the raw noisy data series into several stable modes, which are then input simultaneously to a light-gradient boosting machine (LightGBM) for easier prediction. Zhou et al. [84] used the VMD and deep learning methods to achieve accurate bus arrival predictions. Experiments showed that VMD improved the forecasting accuracy of near-term bus link speed. Furthermore, Shi et al. [85] optimized the selection of parameters in VMD, such as the number of modes, using a scalable artificial bee colony (SABC) algorithm for network traffic flow prediction.

### 3.5. Singular Spectrum Analysis

Another frequency-based decomposition method is singular spectrum analysis (SSA) [86,87], which has received relatively less attention but still shows promising application prospects from the published literature [88].

Zhang et al. [89] experimented with traffic flow forecasting in Houston, Texas, and proposed a multistep ahead forecasting method that decomposes the original data into three individual modeling components: an intraday or periodic trend, a deterministic part, and a volatility part. The three components with different physical meanings were modeled using the spectral analysis strategy, ARIMA, and the GJR-GARCH model (generalized autoregressive conditional heteroskedasticity with a conditional variance formulation), respectively. Their experimental results provide valuable insight into the underlying periodic characteristics and volatile nature of traffic flow data. Based on [89], Zhang et al. [90] decomposed the traffic flow data from freeway I-694 EB in the twin cities into four different components, namely a periodic, trend, stationary, and volatility part, to further explore the underlying traffic patterns. The approaches proposed in [89,90] have notable advantages over general data-driven methods (e.g., NNs, usually black boxes) for interpreting the intrinsic characteristics of traffic data.

Furthermore, Guo et al. [91] used SSA as a smoothing strategy to improve the accuracy of short-term traffic flow predictions. The performance of SSA was evaluated under both normal (non-incident) and abnormal (incident) traffic conditions using data from a corridor in Central London. Kazemi et al. [92] employed the recursive SSA technique to process traffic state data in an online manner and used denoised traffic state measurements for microscopic time-variant car-following behavior simulation. A practical validation of real-world traffic data collected at the Hollywood freeway section of the US 101 highway was conducted.

Xiao et al. [93,94] established hybrid models based on SSA and deep learning technologies for air transport demand forecasting in Hong Kong. In [93], SSA functioned to identify and extract the trend and seasonality properties of air transport demand, while deep learning technologies, including adaptive-network-based fuzzy inference system (ANFIS) and improved particle swarm optimization (IPSO), were responsible for modeling and forecasting the uncertainty and volatility in demand. In [94], the original air passenger volume sequence was first decomposed into three components: trend, seasonal oscillations, and an irregular component, which were then modeled by a generalized regression neural network (GRNN) and radial basis function networks (RNFNs), respectively.

Barba et al. [95,96,97] utilized the singular value decomposition (SVD) of the Hankel matrix (HSVD), which is a variant and one of the steps in SSA, to assist in the establishment of traffic accident prediction models. In [95], HSVD was used to smooth the time series of injured people in traffic accidents in the Chilean region, while the ARIMA and ANN models were adopted for prediction. In [96], the authors proposed a simplified form of SSA combined with AR and ANN methods for accurate traffic accident forecasting in Santiago de Chile. In [97], the traffic accident prediction method is based on the multilevel singular value decomposition (MSVD) of a Hankel matrix. The authors also compared the performance of MSVD with SWT (mentioned in Section 3.2.2) on high and low-frequency components extraction.

The traffic state evolves with strong correlations over both time and space, and fully exploiting these spatiotemporal correlations has become a focus of increasing research. Zhu et al. [21] demonstrated how multi-channel singular spectrum analysis (MSSA) could be utilized to unearth spatiotemporal patterns to iteratively estimate traffic speed conditions over a large traffic network in Shanghai, China, especially when they cannot be directly observed or calculated. Chen et al. [98] introduced truncated SVD for intrinsic spatiotemporal traffic speed pattern extraction and applied SVD-combined tensor decomposition (STD) for robust missing data recovery. Hassan et al. [99] proposed an anomaly detection scheme based on MSSA applied in ITSs. This scheme was designed to extract spatiotemporal properties through traffic speed data streams collected from different highway ramps across the greater Toronto area in Canada.

More reports on the application of SSA to traffic data smoothing, noise reduction, and pattern extraction can be found in [100,101]. The reported studies point to the great potential of SSA for traffic pattern recognition and short-term traffic forecasting.

### 3.6. Other Strategies

In addition to the above decomposition strategies, other alternatives have also been reported in the literature and have yielded positive results in practical applications. In this section, we introduce two other decomposition strategies: STS and STL.

#### 3.6.1. Structural Time Series

As a special time-series analysis technique, STS models treat complex traffic variables (such as flow and speed) as superpositions of various components with different interpretations (such as trends, cycles, seasons, and irregular components). A general univariate STS model for a traffic variable y can be expressed as Equations (1)–(3), involving all possible types of physical components:

(1) $$y_{i}=\mu_{i}+\varphi_{i}+\gamma_{i}+\varepsilon_{i},\;\varepsilon_{i}∼NID(0,\sigma_{\varepsilon }^{2})$$

(2) $$\mu_{i+1}=\mu_{i}+\beta_{i}+\eta_{i},\;\eta_{i}∼NID(0,\sigma_{\eta }^{2})$$

(3) $$\beta_{i+1}=\beta_{i}+\delta_{i},\;\delta_{i}∼NID(0,\sigma_{\delta }^{2})$$

where $y_{i}$ is the observed target traffic variable at time i and $\mu_{i}$, $\varphi_{i}$, $\gamma_{i}$, and $\varepsilon_{i}$ denote the trend, seasonal, cycle, and random error components, respectively.

The STS method provides a clear and flexible description of complex variables. Thus, it is easier to accurately express the dynamic characteristics of a sequence over time. The powerful representation capability of STS enables the original traffic sequence to be decomposed into different components for separate modeling.

Ghosh et al. [102] proposed a multivariate STS method and applied it to short-term urban traffic flow prediction. Different components of the original traffic dataset, such as trend, seasonal, cyclical, and calendar variations, were modeled and analyzed separately using the STM method. A case study of severe traffic congestion in central Dublin, Ireland, demonstrated that the proposed STS model can accurately predict real-time traffic flow at multiple intersections within an urban transport network. Junus et al. [103] studied the behavior of road accidents in Peninsular Malaysia using the STS approach. Experimental results showed that different unobserved components can be separated by the STS method for individual modeling. De Nailly et al. [104] decomposed the data of daily incoming flows of passengers to two transport lines in the Parisian public transport network. A better interpretation of the Paris traffic patterns was obtained from the decomposed data.

#### 3.6.2. Seasonal and Trend Decomposition Using LOESS

Another common alternative decomposition model is seasonal and trend decomposition using LOESS (locally estimated scatterplot smoothing) or STL. STL can be viewed as a filtering procedure without mathematical theoretical assumptions that easily decomposes time-series data into additive variation components, that is,

(4) $$y_{i}=t_{i}+s_{i}+r_{i}$$

where $y_{i}$ is the observed target traffic variable at time i and $t_{i}$, $s_{i}$, and $r_{i}$ denote the trend, seasonal, and remainder components, respectively.

Zhu et al. [105] used STL to decompose taxi trip data for over seven years in Manhattan (New York City) and detected traffic events from the decomposed remainder component. The experimental results show that STL can effectively reveal the dynamic characteristics of urban travel patterns and identify events. Zhou et al. [106] adopted the STL method to forecast the taxi demand. Three simpler components were modeled using different forecasting methods and integrated with the optimal weights obtained by a genetic algorithm (GA). Qin et al. [107] decomposed air and railway passenger flow data from China into seasonal, trend, and remainder components using the STL approach and employed an improved ESN and seasonal-naive method to forecast the trend, remainder, and seasonal component, respectively. Zhao et al. [108] performed a similar study in which the STL method was introduced to assist the prediction of short-term subway passenger flow.

### 3.7. Summary

The various decomposition strategies have their advantages and disadvantages. WT and EMD are currently the most widely discussed strategies for short-term traffic state forecasting. However, in practical applications, modelers must choose according to the characteristics of the research problem and observed data. Table 1 provides a simple comparison of different decomposition strategies.

## 4. Discussion

Although the successful application of the DR-based framework in short-term traffic state forecasting has been demonstrated, several relatively under-conceived challenges still exist. Which DR strategy performs best? How do we determine the optimal feature subset and suitable basic predictors? How can a DR program be executed in practical applications? How can a model’s interpretability be enhanced? In this section, we focus our discussion on the following issues: comparison of decomposition strategies (Section 4.1), feature selection (Section 4.2), determination of basic predictors (Section 4.3), possibility of involving future unknown information (Section 4.4), identification of travel patterns and enhancing model interpretability (Section 4.5), applications in different domains (Section 4.6), and improvement for benchmark non-decomposition models (Section 4.7).

### 4.1. Comparison of Decomposition Strategies

As mentioned above, because different decomposition strategies can improve the performance of basic short-term traffic state predictors, a natural question arises: Which decomposition method contributes the most to the degree of performance improvement? Several researchers have compared the performance gains of different decomposition strategies [25,51,109,110,111,112]. For example, in [112], EEMD outperformed DWT and EMD when using SVM for short-term traffic flow prediction, whereas in [25], FT combined with the A-CFS method had an advantage over the DWT and EEMD methods. Furthermore, SD-based strategies were considered in other studies to provide more detailed information and thus outperform single strategies [22,23]. However, there is no consensus in the existing literature on this issue. Therefore, the authors suggest that future research should provide insights into the selection of different decomposition strategies. Note that this proposal is not intended to derive an optimal policy for all problems and scenarios, which is impossible according to the so-called “No Free Lunch Theorem” [113]. There is no “super algorithm” that works best for all problems. The purpose of this proposal is instead to provide a general analytical framework for different questions.

### 4.2. Feature Selection

As previously mentioned, not all components must be input into the basic predictors. Feature selection aims to find an optimal feature subset by eliminating irrelevant or redundant features (i.e., components obtained by decomposition strategies), thereby improving the accuracy and reducing the computational costs. In contrast, the selection of truly relevant or meaningful features simplifies the model to help understand the process of data generation (interpretability). This demonstrates the importance of feature selection.

From the published works of literature, several methods have been developed to select the appropriate feature subsets for the DR-based short-term traffic state prediction framework. Table 2 summarizes these methods. Pearson’s correlation coefficient (PCC) is one of the simplest methods to help understand the linear relationship between features (decomposed sub-series) and response variables (original series) and is thus adopted in pieces of literature [26]. However, PCC is only sensitive to linear relationships, and therefore cannot be fully used as the basis for the selection of an optimal feature subset. Other methods, including ACF and PE, have been adopted, but are not entirely reliable. Therefore, the authors suggest the development of more sophisticated feature selection engineering.

### 4.3. Determination of Basic Predictors

The performance of a DR-based short-term traffic state prediction framework is largely dependent on the selection of basic predictors. From the published literature, most studies have adopted data-driven methods, especially NNs with deep structures. However, a shared view is that the best prediction performance is achieved when the basic predictor is determined according to the characteristics of the decomposed components, for example, selecting a statistical model such as ARIMA for the linear stationary components. In addition, numerous researchers have held that a hybrid framework combining both statistical and data-driven predictors can enhance stability and interpretability. Therefore, the authors suggest that future research should focus more on analyzing the characteristics of components and provide advice on the selection of appropriate basic predictors for components with different characteristics.

### 4.4. Possibility of Involving Future Unknown Information

Although DR-based prediction frameworks have been reported, a common flaw that has been overlooked by many researchers is that DR frameworks are prone to involving unknown future information. Suppose a traffic parameter time series is $V=\{V_{train},V_{test}\}=\{V_{1},V_{2},\ldots ,V_{A},\ldots ,V_{A+B}\}$, where $V_{train}=\{V_{1},V_{2},\ldots ,V_{A}\}$ is used for a model’s parameters calibration, and $V_{test}=\{V_{A+1},V_{A+2},\ldots ,V_{A+B}\}$ is used for verifying a calibrated model’s performance. In most studies, in order to compare the prediction accuracy of DR-based models with benchmark non-decomposition methods, the entire dataset V is used for decomposition. In this way, the test set $V_{test}$ involving future information contributes to both the decomposition process and training process, which makes the comparison unfair.

For practical applications, the authors suggest that the DR-based hybrid framework can be implemented in the following two ways. The first method is to execute the entire decomposition procedure step by step as new samples become available. This method is feasible but computationally inefficient. Another method is to perform recursive algorithms, such as the recursive SSA proposed by Mirmomeni et al. [114] for dynamic and online applications.

### 4.5. Identification of Travel Patterns and Enhancing Model Interpretability

DR-based hybrid frameworks provide a platform for identifying the underlying travel patterns. From the published works of literature, most studies have focused on the identification of temporal travel patterns, such as trends, cycles, fluctuations, and others. However, there are few existing studies on the development of spatial correlations, which makes sense for the construction of short-term traffic state evolution models, especially at the city-wide or network-wide level; therefore, there is a need for further studies in this area. The related literature provides sufficient evidence to support the idea that the incorporation of spatial correlations can enhance short-term traffic state prediction [115,116,117]. However, properly capturing these correlations is difficult because they typically do not follow a simple distance rule. We expect that DR procedures based on cross-sectional (cross-traffic link) data may be helpful in discovering spatial correlations. Researchers can analyze the correlation coefficients from the extracted unobserved components. This idea, however, has not yet been reported in the literature.

On the other hand, the identified spatiotemporal correlation patterns help improve the explanatory power of the forecasting models; for example, one can understand urban commuting travel patterns (e.g., peak phenomenon of passenger flow and differences in travel characteristics between weekdays and weekends) from the extracted trends and cycles. Although some achievements have been made, there is still room for improvement in this field. Therefore, the authors suggest that future research should consider not only the improvement of the accuracy but also the explanatory power when developing a predictive model.

### 4.6. Applications in Different Domains

As mentioned above, DR procedures have been widely used by researchers in different fields for various forecasting tasks, such as traffic flow, speed, and travel time forecasting for highways or urban roads, air or rail passenger demand forecasting, and metro passenger volume forecasting. Simply put, DR procedures provide a general and rich framework that researchers can use to improve basic predictive models. The versatility and generality of this framework makes its applications not limited to the field of traffic engineering; it is also suitable for prediction tasks in other counting applications, such as wind speed forecasting. However, it is important to mention that one of the most important commonalities of this type of forecasting task is that the data used for forecasting can be seen as a superposition of various components with different characteristics (such as high/low frequency, trends, or cycles) that may be extracted for separate analysis. For example, the daily air passenger demand has both a seasonal cycle and a year-over-year trend. If the prediction object is not composed of different components, then the DR procedure may not be applicable. Table 3 provides a brief summary of the applications in each domain. From the published literature, EMD and WT are the most popular and widely used strategies in various applications.

### 4.7. Improvement for Benchmark Non-Decomposition Models

DR procedures have been widely reported to enhance the basic predictive models for short-term traffic state forecasting. This section provides quantitative information for improving the prediction accuracy of different DR strategies, as shown in Table 4. The prediction accuracy of the decomposition methods was measured using the mean absolute percentage error (MAPE), formulated as

(5) $$MAPE=\frac{1}{n}\sum_{t=1}^{n}\left|\frac{y_{t}-\hat{y}i}{y_{t}}\right|$$

where yi and $\hat{y}i$ denote the actual and predicted values at time i, respectively, and n denotes the number of samples.

Note that this information was collected from [25,51,109,111,112], in which at least two different decomposition strategies were used for comparison. Quantitative comparisons across the literature were not considered in this study. The reasons are twofold. First, the datasets used by various applications are different. Generally, they are aggregated at 15 min granularity for short-term traffic flow, speed, and travel time forecasting of highways or city roads [17,19,24,27,28,37,38,40,41,42,43,44,70,71,81,82]. In applications of bus speed or travel time prediction, they are aggregated at 5–30 min granularity [47,48,55,62,83,101,108]. In applications of air or railway passenger demand prediction, the granularity is usually greater than 24 h [22,30,66,80,93,94,102,107]. Second, different optimization techniques (such as GA [106] and the bat algorithm [81]) have been adopted in these applications, which make different efforts to optimize the basic models and thus may not facilitate a fair comparison of different DR programs.

From the results of [25,51,109,111,112], WT and EEMD showed good performance in most cases, whereas FT outperformed WT and EEMD only when used with other tricks. Although these works have studied the performance improvement of different DR programs, more comparisons are required. More importantly, the authors suggest conducting experiments on publicly available standard datasets to further explore the degree of performance improvement of different DR procedures.

## 5. Conclusions

This study surveyed recently reported DR-based frameworks for short-term traffic state forecasting, focusing on how decomposition and reconstruction strategies can be utilized to enhance the predictability and interpretability of basic predictors of traffic variables, including flow, speed, travel time, passenger/freight volume, travel demand, and even accidents. Specifically, we first introduced the general procedures of DR-based hybrid frameworks; thereupon, the applications of decomposition strategies, including FT, WT, EMD, VMD, SSA, STL, STS, and some of their variants in short-term traffic state prediction were reviewed. Furthermore, several challenges and possible future directions for the application of these DR-based procedures were discussed.

The review and discussion in this paper enable researchers to gain a comprehensive understanding of the current state of short-term traffic state prediction based on the DR framework, thereby avoiding duplication of effort. The contribution of this work also includes providing a reference for modelers to choose appropriate decomposition and reconstruction strategies and some insights for future research.

## Figures and Tables

**Table 1:** Comparison of various decomposition strategies.

Decomposition Strategy	Pros	Cons	References
FT	Clean and broadband frequency spectrum	Stationarity assumption	[20,25,31,33,34,35,36]
WT	Simultaneous and multiresolution analysis of both time and frequency	Manual selection of basis wavelet and decomposition level	[17,19,23,24,29,38,39,40,41,42,44,45,46,47,48]
WPT	Provides more detailed information	Same as WT	[22,50]
SWT	Translation invariance	Same as WT	[37,43,53]
EMD	Adaptive	End effect; modal aliasing; sensitivity to noise and sampling	[26,55,56,57,58,59,60,61,62,67]
EEMD	Adaptive; suppress mode aliasing	Relatively high reconstruction error and computational cost; poor decomposition completeness	[27,28,30,64,66,68,72,73,74,75]
CEEMDAN	Adaptive; almost no additional noise in the reconstructed signal	Higher computational cost	[70,71]
VMD	Effectively suppress modal aliasing; robust to sampling and noise	A predefined number of modes	[14,79,80,81,82,83,84,85]
SSA	Widely applicable	Manual determination of a few parameters	[21,22,89,90,91,92,93,94,95,96,97,98,99,100,101]
STL	Widely applicable and flexible	Same as SSA	[105,106,107,108]
STS	Same as STL	Homoscedasticity assumption of residuals	[102,103,104]

**Table 2:** Summary of adopted feature selection methods.

Feature Selection Methods	References
ACF (autocorrelation function)	[37,40,50,80]
FASTNet (frequency-aware spatio-temporal network)	[33]
A-CFS (adaptive cutoff frequency selection method)	[25]
PCC (Pearson product moment correlation coefficient)	[26]
KCC (Kendall rank correlation coefficient)	[26,62]
SCC (Spearman correlation coefficient)	[62]
MRMR (minimum redundancy maximum relevance)	[27]
PE (permutation entropy)	[61,70]
AE (approximate entropy)	[28,108]
IWPE (improved weighted permutation entropy)	[71]
PSR (phase space reconstruction)	[82,100]

**Table 3:** Summary of applications in various domains.

Transportation Modes	Parameters ^1^	References					
		FT	WT	EMD	VMD	SSA	STL/STS
Highway/urban road	Flow	[25,33,34,35]	[17,19,24,29,37,38,40,41,42,43,44,50,53]	[27,28,63,67,70,71,73,74,75,76]	[79,81,82,85]	[89,90,91,92,100]	[102]
	Speed	-	[39,46]	[56,57,58,59,72]	[14]	[21,98,99]	-
	Travel time	[36]	[45]	[68]	-	-	-
Metro	Passenger volume	-	[47,48]	[26,55,62]	[83]	[101]	[108]
Bus	Speed/travel time	-	-	[60]	[84]	-	-
Aviation	Passenger demand	-	-	[66]	[80]	[93,94]	[107]
Railway	Passenger demand	-	[22]	[30]	-	[22]	[104,107]
Others	-	[20,31]	-	[61,64]	-	[95,96,97]	[103,105,106]

^1^ Parameters refer to the predicted traffic state variables.

**Table 4:** Degree of performance improvement.

References	Application	Dataset	Baseline	Prediction Accuracy ^2^ (Degree of Improvement)				
				BL ^1^	FT	WT	EEMD	VMD
[25]	Traffic flow forecasting of highway	England National Highways ^3^	KF	0.1078	0.0875 (+18.8%)	0.0900 (+16.5%)	0.0896 (+16.8%)	-
[51]		PeMS ^4^	LSTM	0.0886	-	0.0306 (+59.1%)	0.0840 (+5.19%)	0.0635 (+28.3%)
[109]		PeMS ^4^	ANN	0.1231	-	0.0520 (+57.7%)	-	-
[111]		PeMS ^4^	LSTM	0.0901	-	0.0246 (+72.6%)	0.0210 (+76.7%)	-
[112]		TDRL ^5^	SVM	0.1118	-	0.0954 (+14.6%)	0.0928 (+17.1%)	-

^1^ BL refers to the baseline non-decomposition models such as KF, LSTM, ANN, and SVM. ^2^ Prediction accuracy is measured by the mean absolute percentage error (MAPE). Degree of improvement (DI) is measured by $DI=\frac{MAPE_{BL}-MAPE_{DR}}{MAPE_{BL}}$, where $MAPE_{BL}$ and $MAPE_{DR}$ denote the MAPEs of the baseline and DR models, respectively. ^3^ Website (highwaysengland.co.uk). ^4^ PeMS (Caltrans Performance Measurement System). ^5^ TDRL (Transportation Data Research Laboratory) in University of Minnesota Duluth.
